# Development of Facial Soft-Tissue Asymmetry During Growth: A Mixed-Longitudinal Three-Dimensional Study

**DOI:** 10.3390/jcm15218583

**Published:** 2026-11-04

**Authors:** Peter Direr, Marta Gibas-Stanek, Robin Bruggink, Petra Kaminkova, Mette Kuijpers, Thomas Maal, Piotr S. Fudalej

**Affiliations:** 1 Institute of Dentistry and Oral Sciences, Palacký University Olomouc, Palackého 12, 772 00 Olomouc, Czech Republic; 2 Department of Orthodontics, Jagiellonian University Medical College, Montelupich 4, 31-155 Kraków, Poland; 3 Radboudumc 3D Lab, Radboud University Medical Center, Philips van Leydenlaan 25, 6525 EX Nijmegen, The Netherlands; 4 Department of Dentistry, Section Orthodontics and Craniofacial Biology, Radboud University Medical Center, Philips van Leydenlaan 25, 6525 EX Nijmegen, The Netherlands; 5 Amalia Cleft and Craniofacial Centre, Radboud University Medical Center, Philips van Leydenlaan 25, 6525 EX Nijmegen, The Netherlands

**Keywords:** adolescent, allometry, child, facial asymmetry, facial growth, imaging, three-dimensional, longitudinal studies, photogrammetry

## Abstract

**Background/Objectives**: Most healthy faces are mildly asymmetric, but how this asymmetry changes during growth and relates to facial size is unclear. We examined whether whole-face soft-tissue asymmetry increases as children grow and whether any increase simply reflects facial growth. **Methods**: We analysed 1014 three-dimensional facial scans of 377 children aged 6.0–18.8 years, followed for up to 3.2 years (median 2.0); the primary asymmetry analysis included 1003 scans of 376 children, after whole-face values with an isolated extreme point were set to missing. Asymmetry was the mean absolute perpendicular distance between the facial surface and its mirror image. Mixed-effects models separated within-child change from between-child differences. Repeat-capture reliability was not assessed. **Results**: Asymmetry increased by 3.2% per year within children entering at 11 years (95% confidence interval [CI], 2.2–4.1); older entrants were 2.7% more asymmetric per year of age (95% CI, 1.6–3.8). After adjustment for imaging session, the estimate was similar but imprecise (3.4%; 95% CI, −1.4 to 8.5). Facial size increased by 1.5% per year and asymmetry relative to size by 1.6%. Within children, asymmetry rose by about 1.7% for each 1% increase in facial size (exponent 1.73; 95% CI, 1.28–2.19); after adjustment for age this association was imprecise (0.89; 95% CI, −0.13 to 1.91), so the contributions of facial size and age could not be separated. Boys had 6.5% greater asymmetry; relative to facial size, 3.1% (95% CI, −2.0 to 8.6). **Conclusions**: On average, facial asymmetry increased modestly as children grew and increased relative to facial size over the observation period; whether this reflects facial enlargement itself or other age-related changes could not be determined. The estimated annual increase (about 0.013 mm) was below the residual variability between scans (about 0.04 mm); these population-level findings suggest that change cannot be detected in individual children, and individual monitoring and diagnostic thresholds require validated measurement.

## 1. Introduction

No human face is perfectly symmetrical. A mild degree of left–right asymmetry is found in practically every healthy individual and in the population as a whole: in large samples the right hemiface is usually wider or more protrusive than the left [1,2,3], and the facial midline tends to form a slightly bent, C-shaped curve [4]. Asymmetry nevertheless matters clinically. Facial asymmetry is a frequent reason for seeking surgical-orthodontic treatment [5] and is relevant to assessment of cleft lip and palate, hemifacial microsomia, unilateral crossbite and facial trauma. However, no generally applicable threshold separates normal from increased asymmetry [6,7]. Age-related reference data may help place facial soft-tissue asymmetry in its developmental context.

Facial asymmetry has several origins. Craniofacial asymmetry is already present in the skulls of fetuses and infants, before a chewing pattern is established [8], and is thought to reflect both heredity and function, such as the side preferred for chewing or sleeping and unilateral crossbite [7,8,9]. During growth, the two sides of the face may also differ in skeletal growth, soft-tissue thickness and prominence, or functional adaptation. Puberty may change the timing of these processes, so the same chronological age need not mean the same developmental stage in girls and boys. Two kinds of asymmetry are usually distinguished: directional asymmetry, a side bias shared by most people, and fluctuating asymmetry, the small individual deviations around this shared pattern [10,11]. The measure used in this study captures the overall amount of surface asymmetry and does not separate the two. Even patients considered clinically symmetric show mild side differences in the mandible, which may be partly compensated by the soft tissues [12]; soft tissues can thus mask part of the underlying skeletal asymmetry [1]. The soft-tissue surface is also what patients and others see, which makes it a relevant focus for assessing development.

Methods for measuring facial asymmetry have changed considerably. Anthropometry and radiographs capture only selected landmarks and distances, and repeated radiographs cannot be justified merely to follow normal growth in healthy children [9]. Three-dimensional surface imaging records the whole face without ionizing radiation. Asymmetry can then be quantified as the distance between the original and the mirrored surface [13] or between paired points of a template fitted to both sides of the face [3,14]. Quantitative photogrammetric measurements show higher interobserver correlations than subjective assessments (r = 0.91–0.95 vs. 0.56) [15]. However, these correlations do not establish the reproducibility of repeated image acquisition and processing. Normative values for whole-face asymmetry in children depend on the facial region analysed, the alignment method and the way distance is defined [3,14,16,17]. Dense correspondence methods sample the facial surface consistently [11], but how well they perform across ages and facial sizes still needs to be validated for each application.

How facial asymmetry changes as children grow remains unresolved. Longitudinal studies show a decrease between 1 and 6 years of age [7,9]. Studies that followed older children found no change in whole-face asymmetry, either between 5 and 10 years [16] or between 11.5 and 16 years [6]. In a mixed-longitudinal study of children with unilateral cleft lip and palate, the control group of 63 children without craniofacial anomalies, aged 6 to 12 years, showed an increase only at the base of the nose [18]. Cross-sectional findings are contradictory. A large pediatric sample of 533 children aged 0 to 18 years showed no association with age [14], whereas other studies report more asymmetry in older than in younger children [17] and a steady increase across the lifespan [19,20]. Developmental biology offers no simple prediction either. The symmetry of the hands increases across childhood [21], as expected if growth tends towards a symmetric optimum, whereas facial asymmetry is also shaped by function and by unequal growth of the two sides.

Two features of the existing evidence [6,7,9,14,16,17,18,19,20] make these findings difficult to reconcile. First, the longitudinal samples are small (27 to 75 children) [6,7,9,16,18], each covers only a few years, and none spans both school age and adolescence. The most recent semi-longitudinal study, of 46 adolescents aged 15 to 20 years, had to analyse its repeated scans as independent cross-sectional groups [22]. Larger longitudinal 3D datasets of facial growth exist [23], but they have not been analysed for asymmetry. Comparisons of different age groups, in turn, cannot show whether individual children become more asymmetric or whether children born at different times simply differ. Second, previous studies have described how asymmetry differs between age groups or changes over time, but have expressed it in millimetres or after scaling all faces to a common size [7]. A larger face with the same proportional asymmetry yields larger distances. To our knowledge, no study has estimated how asymmetry changes relative to facial size within growing children, that is, its longitudinal allometry. In geometric morphometrics, the relation between size and shape (allometry) is analysed explicitly [24], and in children, age and different measures of size are associated with partly different aspects of facial shape [25]. Size also matters for the frequent finding that boys and men are more asymmetric than girls and women [17,20], because males have larger faces with more prominent noses and mouths [26].

We therefore used a mixed-longitudinal sample of children aged approximately 6 to 19 years, imaged annually up to four times, to examine both how asymmetry changes within individual children over a few years and how it differs between children of different ages. Our aims were (1) to estimate within-child change in whole-face asymmetry and compare it with differences between children of different ages; (2) to examine whether asymmetry changes in proportion to facial size; and (3) to assess differences between boys and girls. Facial size was measured as centroid size, an overall measure calculated from the same dense set of points used to measure asymmetry. Unlike facial height or width, it summarizes the whole face rather than a single dimension, but it cannot show where the face grows. We hypothesised that asymmetry would increase with age, but that this increase would largely reflect facial growth.

## 2. Materials and Methods

### 2.1. Study Design and Participants

This mixed-longitudinal study used 3D facial images collected as part of a school-based longitudinal project in the Olomouc region of the Czech Republic. At the start of the project, 10 primary and secondary schools were invited to participate and four agreed to take part. Detailed information about the study and consent forms were provided to families through the participating schools. The number of eligible children in the participating schools, the number of families approached and the reasons why six schools declined were not recorded; the flow of scans is shown in Figure S1. No a priori sample-size calculation was performed; the analysis used all available scans from the cohort.

#### 2.1.1. Inclusion Criteria

Participants were required to be in good general health and to have two parents who were native speakers of Czech. This criterion was used as a practical proxy for a homogeneous Central European background, to reduce population heterogeneity in facial morphology and to describe growth within a single study population; it does not establish genetic ancestry. Participation required parental or guardian informed consent and child assent, as described in Section 2.2.

#### 2.1.2. Exclusion Criteria

Exclusion criteria were congenital craniofacial anomalies, previous craniofacial trauma, clinically pronounced skeletal malocclusion (a clinical judgement at the screening examination of markedly retruded chin or visible facial asymmetry; the number of children excluded on this ground was not recorded) and previous orthodontic treatment. At the entry visit, a basic orthodontic examination recorded Angle classification and other clinically relevant orthodontic findings, including teeth in crossbite; less severe malocclusions, including unilateral posterior crossbite, were not exclusion criteria. Under the study protocol, children who started orthodontic treatment were not eligible for subsequent observations. This rule ended participation during follow-up; it differs from the exclusion criteria applied at entry and from the loss of individual scans for technical reasons.

#### 2.1.3. Observation Schedule

Children were recruited in four school cohorts and imaged at intervals of approximately one year in 11 sessions between December 2016 and February 2020. The first cohort (166 children) was imaged in December 2016, December 2017, February 2019 and February 2020; two cohorts (111 and 77 children) were imaged three times in November or February; and the smallest cohort (23 children) was imaged three times in April or May. Further planned sessions were cancelled because of the COVID-19 pandemic, so children contributed up to four observations. Children entered at different ages, so overlapping short individual trajectories covered approximately 6 to 19 years at the population level. No child was followed continuously across this full age range. Data from part of this sample have been reported previously [27,28].

### 2.2. Ethics

The study was conducted in accordance with the Declaration of Helsinki and approved by the Bioethics Committee of Palacký University Olomouc, Olomouc, Czech Republic (reference no. 69/15). The approved protocol covered repeated three-dimensional facial imaging and its use in research on facial growth. Written informed consent was obtained from a parent or legal guardian for each participant, and participating children provided assent.

### 2.3. Three-Dimensional Facial Imaging

Facial images were acquired with a VECTRA M3 three-dimensional stereophotogrammetric optical scanner (Canfield Scientific Inc., Fairfield, NJ, USA) by two experienced operators. The system uses six synchronized cameras to capture the facial surface simultaneously from multiple viewpoints in approximately 3.5 ms and reconstruct a photorealistic 3D model while preserving facial geometry. The scanner was calibrated at the start of each imaging session in accordance with the manufacturer’s instructions. Calibration records and session-level acquisition conditions were not archived, so systematic differences between sessions could be examined only statistically (Section 3.4). The same two operators used the same equipment and followed a standardized acquisition protocol throughout 2016–2020.

**Figure 1:**
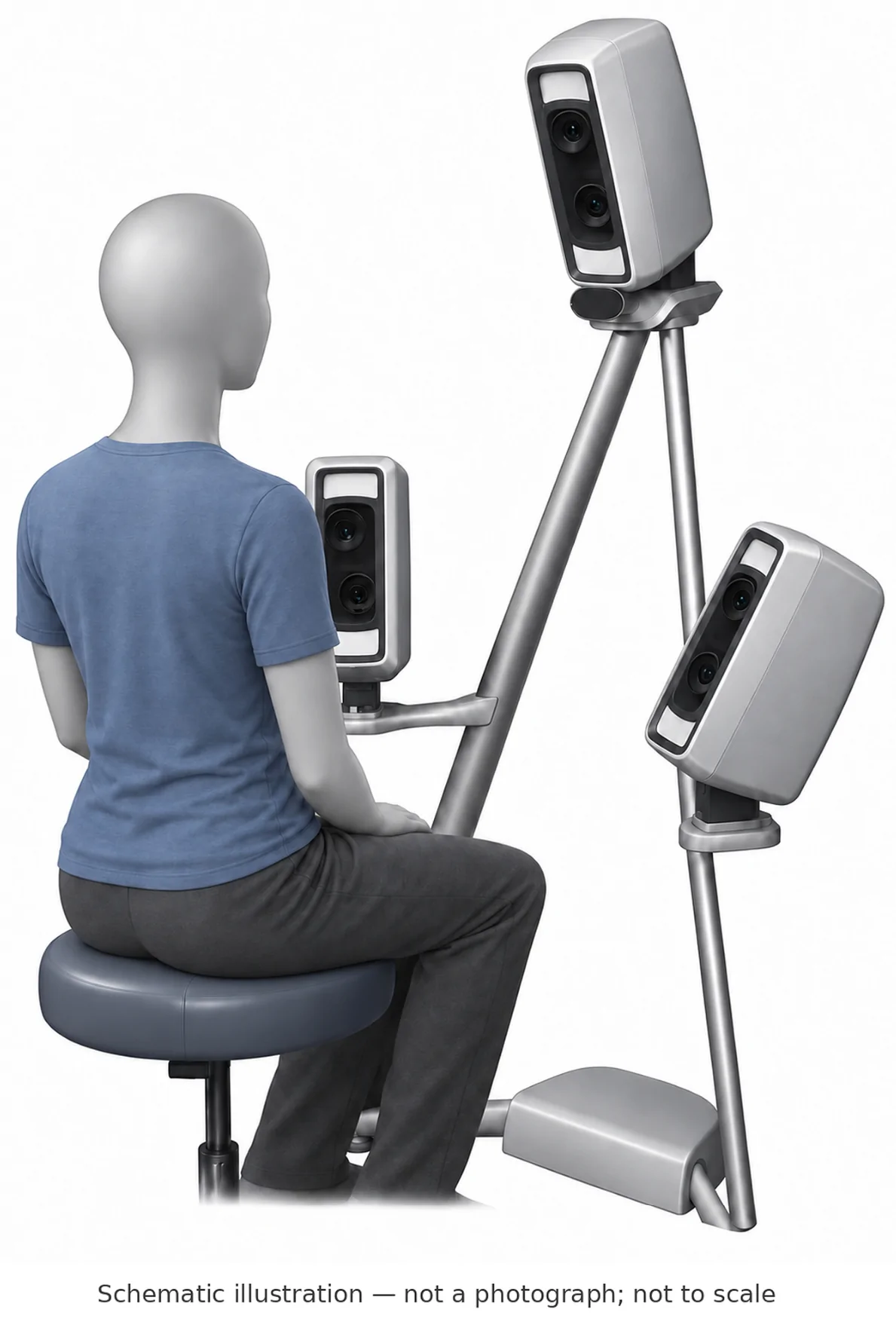
Schematic illustration of three-dimensional image acquisition with the VECTRA M3 system. The participant sits in one position in front of the camera units, which capture the face simultaneously in a single exposure (approximately 3.5 ms); no repositioning between views is needed. The illustration was created with the help of a generative AI tool (ChatGPT v.5.6, OpenAI (CA, USA)). The figure is not a photograph and is not to scale; the resulting facial surface and its processing are shown in Figure 2.

**Figure 2:**
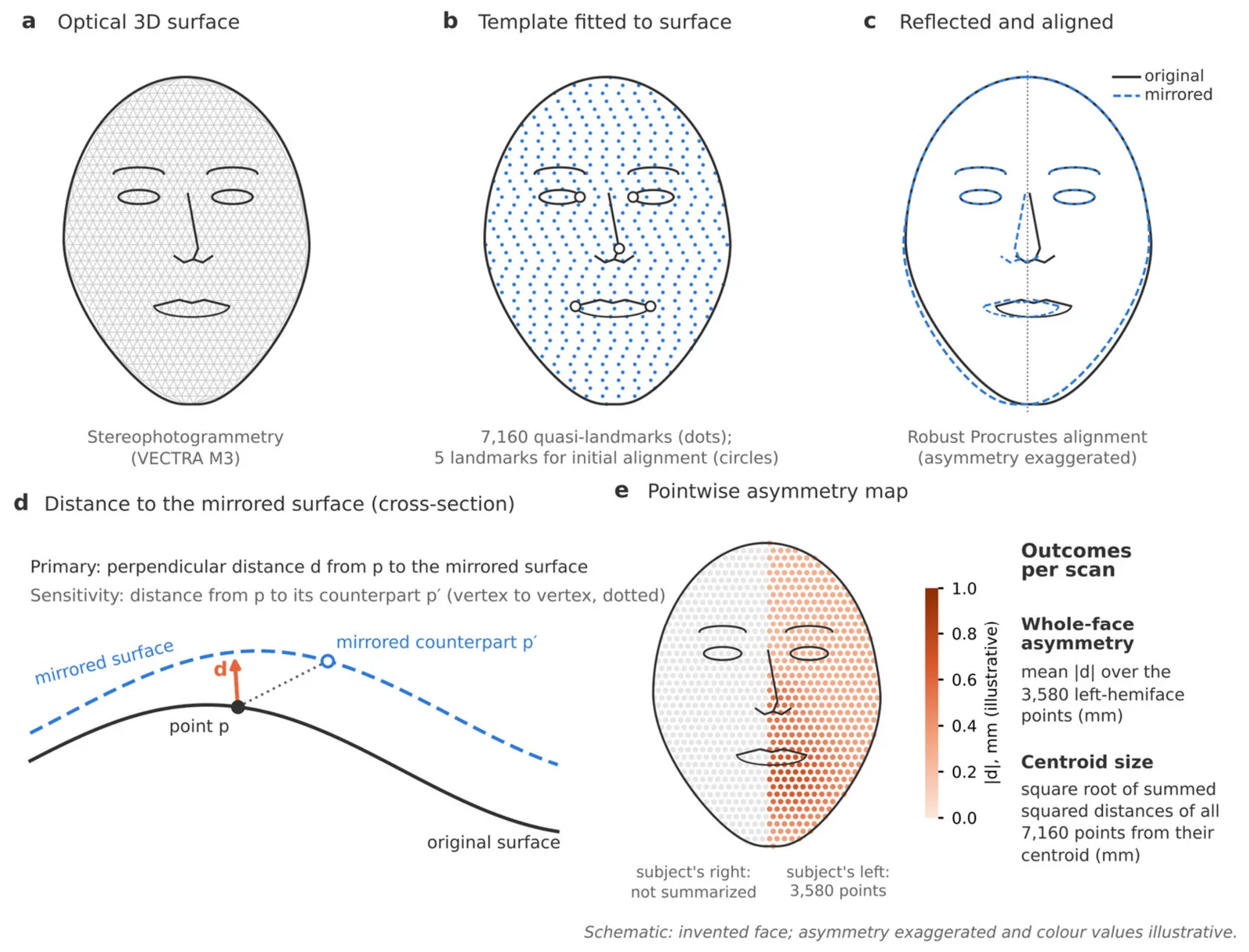
Schematic of the measurement workflow. (**a**) Facial surface acquired by stereophotogrammetry. (**b**) A symmetric template of 7160 quasi-landmarks is fitted to each surface after initial alignment on five landmarks. (**c**) The fitted template is reflected and aligned with the original by robust Procrustes registration. (**d**) At each point p, asymmetry is the perpendicular distance d to the mirrored surface; the sensitivity analysis used the distance from p to its mirrored counterpart p′. (**e**) Pointwise distances are summarized as the mean absolute distance over the 3580 points of the subject’s left hemiface; centroid size is computed from all 7160 points. The face is schematic, asymmetry is exaggerated and colour values are illustrative.

Participants were seated on an adjustable chair facing a mirror positioned between the camera units. Head position was standardized using the alignment guides displayed by the acquisition software. During image capture, participants were asked to remain still, maintain a neutral facial expression, and keep their teeth in maximal intercuspation. Hair was moved away from the face, with a headband used when necessary. The seated acquisition position is illustrated schematically in Figure 1; the resulting three-dimensional surface and its processing are shown in Figure 2. Acquisition dates were verified in the VECTRA database. Repeated captures were not taken, so the repeatability of image acquisition could not be quantified. Image processing was fully automatic, so processing the same image again gives the same result; this does not, however, show that the measurements are accurate or that a new capture of the same face would give the same value.

### 2.4. Evaluation of Facial Asymmetry

The complete processing workflow was performed automatically in 3DMedX^®^ (version 1.2.49.0; 3D Lab, Radboud University Medical Center, Nijmegen, The Netherlands). It comprised surface standardization and asymmetry quantification. A single symmetric facial template containing 7160 quasi-landmarks, assigned to predefined facial regions, was fitted to each surface across all ages and both sexes. This maintained a common sampling configuration across observations. Template-based dense correspondence of this kind has been used to assess facial growth in children [29,30].

Template fitting was performed automatically with the MeshMonk non-rigid registration framework [31], using a multistage procedure. First, the template was coarsely aligned by Procrustes registration using five automatically identified facial landmarks: the bilateral endocanthia, bilateral cheilions, and pronasale. Second, rigid alignment was refined using an iterative closest point (ICP) algorithm. Third, non-rigid registration was used to deform the template towards the individual facial surface. Finally, all template points were projected onto the facial surface, yielding a common quasi-landmark configuration intended to represent anatomical correspondence across images [29,30].

After standardization, a copy of the fitted template was reflected across its sagittal plane. The reflected and original templates were aligned using a dedicated Procrustes-based registration procedure [32] designed to reduce the influence of asymmetric regions on alignment. Pointwise differences were calculated in millimetres and could be visualized as colour-coded distance maps (Figure 2). The analysis used the 3580 quasi-landmarks of the left hemiface to summarize discrepancies between the original and mirrored representations.

At each quasi-landmark, asymmetry was measured as the perpendicular distance to the mirrored facial surface. This point-to-surface distance is not necessarily the same as the straight-line distance between matching points on the left and right sides. It mainly reflects differences in how far the surface projects and may be less sensitive to points displaced along the surface. Because absolute values were averaged, the measure does not show which side is more prominent and does not separate directional from fluctuating asymmetry.

Whole-face asymmetry was summarized as the mean absolute distance across the 3580 left-hemiface quasi-landmarks, in millimetres. Each point counts equally, so the result depends on how the template points are distributed over the face; it is not an area-weighted average. Facial size was measured as centroid size, the square root of the summed squared distances of all 7160 template points from their common centre. Centroid size is expressed in millimetres but is not a facial length, and its value depends on the number and arrangement of points. Values can therefore be compared only between studies that use the same template, scaling and facial coverage.

### 2.5. Data Checks

Of 1038 scans with acquisition dates verified in the VECTRA database, 23 could not be measured because of defective meshes (missing facial areas or overlapping hair). Linkage involved two steps. First, scans were linked to participants by name, date of birth and acquisition date, using the VECTRA database, in which each scan is stored with the participant’s name and acquisition date, and the assessors’ log of names, dates of birth, scan dates and clinical data; names were then replaced by study codes. Second, the exported symmetry results, which were identified by participant code and by the ordinal number of the processed scan but not by acquisition date or a unique scan identifier, were linked to the dated scans. Results were numbered in chronological order of the processed scans, as confirmed by the processing laboratory, and were linked in the same order within each child; this rule matched the number of results to the number of processed scans in every child. This second step could not be verified independently through unique scan identifiers. Additional uncertainty arising from incomplete processing affected 23 children with at least one unprocessed scan; restricting the analysis to children with complete series gave similar results (Table S1), and facial-size growth between consecutive linked scans was similar in children with and without an unprocessed scan. Ten scans that failed initial processing were processed again; one remained unusable because only part of the face had been captured. In total, 24 scans were excluded during technical processing, leaving 1014 scans for analysis.

In 11 retained scans, a single point lay more than 10 mm from the mirrored surface. Visual inspection (P.D.) confirmed that these isolated values did not correspond to facial features; they most likely reflected holes in the mirrored surface. For these scans, the whole-face asymmetry value was set to missing, leaving 1003 observations for the primary analysis. The same rule (any point beyond ±10 mm) was applied to all scans in both distance outcomes and flagged 12 scans: 11 in the perpendicular-distance outcome and 1 only in the vertex-to-vertex outcome. It was a technical screening rule, not a diagnostic threshold for facial asymmetry. Sensitivity analyses examined other ways of handling these values (Table S1).

### 2.6. Statistical Analysis

Asymmetry and facial size (centroid size) were analysed on a logarithmic scale, so that change is expressed in relative terms (per cent) rather than in millimetres. Results are reported as percentage changes or percentage differences with 95% confidence intervals (CIs). Whole-face asymmetry was the outcome. The full model specification and technical details are given in Supplementary Methods S1.

*Change in asymmetry with age.* Children were scanned up to four times, and not all equally often. We therefore used linear mixed-effects models, which use every available scan and allow each child to have their own starting level of asymmetry and their own rate of change. The model included age at the first scan (centred at 11 years), years since the first scan, their interaction, and sex. This design separates two questions: how much asymmetry changes as each child grows (within-child change), and how much more asymmetric children are when they enter the study at an older age (between-child difference). The interaction tested whether the yearly rate of change depended on the age at entry, and a sex-by-time interaction tested whether boys and girls changed at different rates. To assess departure from the specified linear age pattern, the primary model was compared with a nested natural cubic spline model using a likelihood-ratio test. A nonsignificant result does not establish a constant rate across the full developmental period. Model assumptions were checked graphically (Figure S2).

*Asymmetry relative to facial size.* A larger face can show more asymmetry in millimetres without being proportionally more asymmetric. We therefore (1) estimated the yearly growth of centroid size; (2) analysed asymmetry divided by centroid size, which assumes that asymmetry grows in proportion to the face; and (3) estimated the actual relation between asymmetry and facial size (allometric exponent), both as each child grew (within-child) and between larger and smaller children (between-child). An exponent of one means that asymmetry grows in proportion to the face (isometry); an exponent above one means that it grows faster than the face. Further models compared boys and girls of equal facial size and, because age and facial size increase together, examined both simultaneously.

*Sensitivity analyses.* The main analyses were repeated with an alternative summary of asymmetry (root-mean-square distance), an alternative distance (vertex to vertex), different handling of isolated extreme values, and in subsets of children (at least two scans; complete measurement series; no crossbite; Angle Class I at every visit). We also estimated yearly change separately by age at entry (<10, 10–12.9 and ≥13 years), adjusted for imaging session, and refitted the model leaving out one child at a time (Table S1). Residual variability was summarized by age band as a model diagnostic, not as an estimate of measurement error.

**Figure 3:**
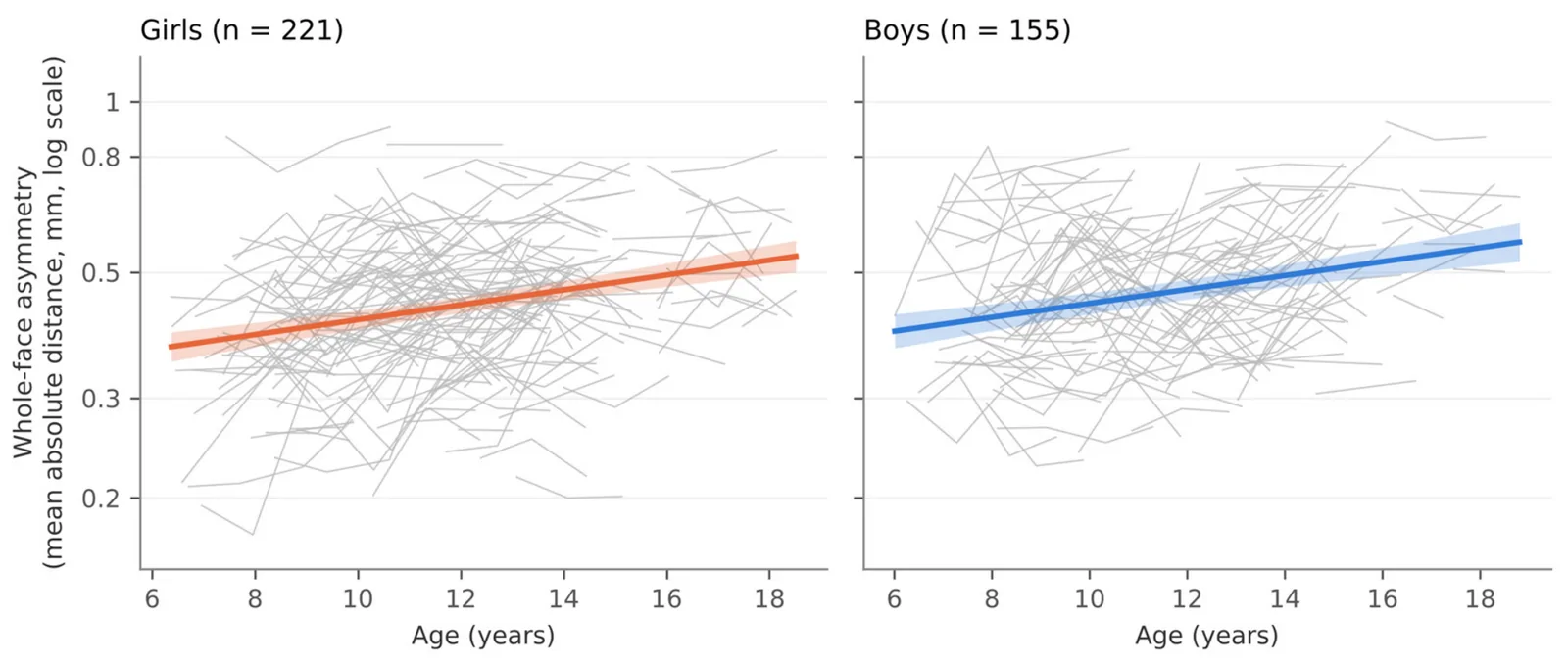
Whole-face asymmetry by age, shown in millimetres on a logarithmic axis. Grey lines connect observations within individuals and cover their observed follow-up only. Coloured lines and shaded bands show the fitted population-level geometric mean from the descriptive age-by-sex model (Section 2.6) and its 95% confidence interval, not individual prediction intervals. Panels show the primary asymmetry sample of 221 girls and 155 boys. Trends across the full age range combine overlapping individual observation periods.

*Missing scans and unmeasured factors.* Reasons for missed visits were not recorded. Participation ended when orthodontic treatment started, but such dropouts could not be distinguished from other absences. The mixed models assume that missed visits are unrelated to the unobserved asymmetry values, given the information in the model; this cannot be verified. Biological maturation was not measured, the operator for individual scans was not recorded, and only left-hemiface outputs were exported. Expected values by age and sex (Figure 3) come from a separate descriptive model (Supplementary Methods S1).

Secondary and sensitivity analyses were interpreted mainly from effect estimates and 95% CIs; two-sided *p*-values are reported without adjustment for multiple comparisons, and *p* < 0.05 was considered statistically significant. Analyses were performed in Python 3.11 (original analyses) and 3.13 (revision analyses) with statsmodels version 0.15.

## 3. Results

### 3.1. Sample

The analytical dataset contained 1014 scans from 377 children (222 girls, 155 boys; Table 1). The primary asymmetry analysis used 1003 scans from 376 children (221 girls and 155 boys, as shown in Figure 3). Mean age at the first scan was 10.8 years (SD 2.6; range 6.0–17.5); ages at imaging ranged from 6.0 to 18.8 years. In the analytical dataset, 337 children (89%) had at least two scans, with a median follow-up of 2.0 years and a maximum follow-up of 3.2 years. The median interval between consecutive scans was 1.00 years. Table 2 reports scans, children and consecutive scan pairs by age band.

### 3.2. Absolute Asymmetry

Median whole-face asymmetry was 0.40 mm at 6–8 years and 0.53 mm at 15–18 years (Table 2). For children entering the study at age 11 years, asymmetry increased by 3.2% per year as they grew (95% CI 2.2 to 4.1; *p* < 0.001; Table 3, Figure 3). In absolute terms this is very small: for a typical value of 0.42 mm, a 3.2% increase corresponds to about 0.013 mm over one year. Children who entered the study at an older age were 2.7% more asymmetric per year of age (95% CI 1.6 to 3.8).

The age-related difference in asymmetry between children was similar to the annual increase observed within individual children (difference −0.5 percentage points, 95% CI −1.9 to 1.0; *p* = 0.53), although this does not prove that the two estimates are equivalent. We found no clear evidence that the annual increase depended on the child’s age at study entry (difference in annual increase per additional year of entry age: −0.03 percentage points, 95% CI −0.41 to 0.35; *p* = 0.88). Allowing the rate of change to vary with age did not improve the model fit (*p* = 0.56). However, these findings do not rule out differences in growth patterns across ages.

Asymmetry increased by an estimated 2.9% per year in children who entered before age 10 (95% CI 1.6 to 4.2; 150 children), 3.5% in those entering at 10–12.9 years (95% CI 2.1 to 5.0; 153 children), and 3.2% in those entering at 13 years or older (95% CI 0.6 to 5.8; 73 children; Table S1). These results suggest a modest increase in all three groups, but do not establish that their rates of change were the same. The estimate for the oldest group was less precise because fewer children were included. Pubertal timing was not measured.

Boys had 6.5% greater absolute asymmetry than girls (95% CI 1.0 to 12.2; *p* = 0.020). There was no evidence of a sex difference in annual change (−0.02 percentage points per year, 95% CI −1.85 to 1.80; *p* = 0.98), although the interval permits differences in either direction.

### 3.3. Facial Size and Size-Relative Asymmetry

Facial size (centroid size) increased by 1.5% per year within children (95% CI 1.4 to 1.6). Boys’ faces were 3.1% larger than girls’ (95% CI 2.3 to 3.9) and grew about 0.25% per year faster (95% CI 0.06 to 0.44; *p* = 0.012). In 43 of 637 consecutive scan pairs (7%), the measured facial size was slightly smaller at the later scan (residual SD on the log scale about 0.68%). Such decreases may reflect image capture, facial expression, registration or genuine soft-tissue change.

**Figure 4:**
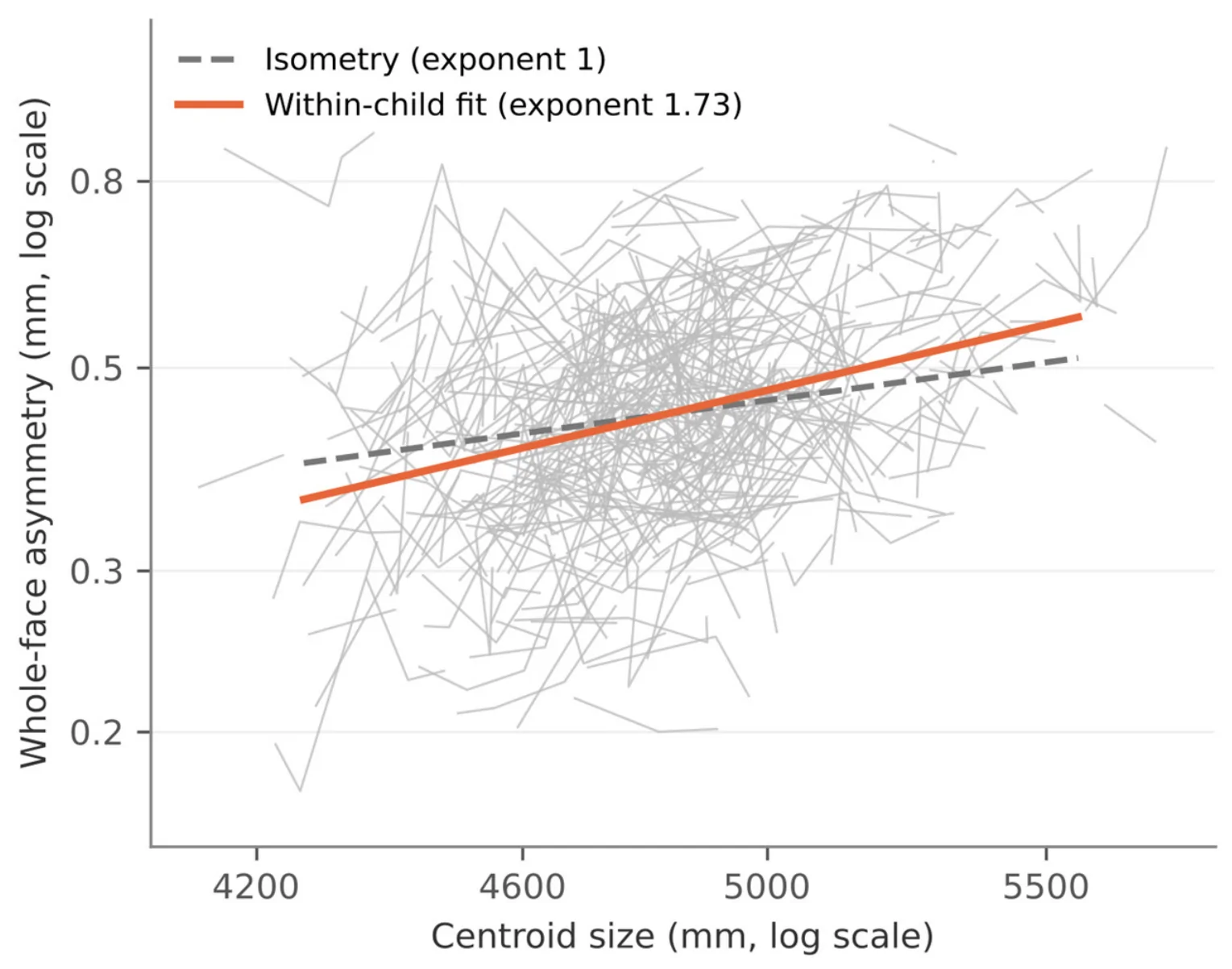
Whole-face asymmetry against facial centroid size, both shown on logarithmic axes. Grey lines connect observations within children. The orange line illustrates the within-child allometric exponent of 1.73 from the model including within-child and between-child log centroid size and sex; the dashed line illustrates an exponent of one (isometry). The allometric model did not jointly adjust for age. Both lines pass through the sample geometric means of asymmetry (0.44 mm) and centroid size (4834 mm). The line represents the estimated within-child association over the observation period and should not be interpreted as an age-independent effect of facial size or as a developmental trajectory; after adjustment for age, the within-child exponent was 0.89 (95% CI −0.13 to 1.91).

Over the observation period, asymmetry increased more than in proportion to facial size. Asymmetry relative to facial size rose by 1.6% per year (95% CI 0.7 to 2.6; *p* < 0.001; Table 4). Correspondingly, each 1% increase in a child’s facial size was accompanied by an increase in asymmetry of about 1.7% (within-child allometric exponent 1.73, 95% CI 1.28 to 2.19; *p* = 0.002 vs. proportional growth; Figure 4), and larger children were more asymmetric than smaller children to a similar degree (between-child exponent 1.69, 95% CI 1.20 to 2.18; *p* = 0.006). Allowing each child their own relation between size and asymmetry gave a similar within-child exponent (1.82, 95% CI 1.29 to 2.35).

The sex difference became smaller once facial size was taken into account. Relative to facial size, boys were 3.1% more asymmetric than girls (95% CI −2.0 to 8.6; *p* = 0.24). In the allometric model, which compares boys and girls of the same facial size, the difference was 0.9% (95% CI −4.3 to 6.3; *p* = 0.75). These wide intervals do not rule out a sex difference, and a smaller difference after adjustment does not show that facial size causes it.

Within children, age and facial size increased together (r = 0.90 after centring time within each child), so their separate contributions could not be estimated precisely (variance inflation factors 3.4 and 3.3 for elapsed time and within-child log centroid size in the joint model; Supplementary Methods S1). When age was added to the size model, the within-child exponent was 0.89 (95% CI −0.13 to 1.91) and the yearly change at constant facial size 1.7% (95% CI −0.1 to 3.5). These analyses therefore show disproportionate scaling during growth: asymmetry increased more than facial size over the same period. They do not establish an association between facial size and asymmetry that is independent of age. Adjusted for both facial size and age, the sex difference was 1.2% (95% CI −4.3 to 7.1).

### 3.4. Sensitivity Analyses

Results were similar across the alternative analyses (Table S1). With different asymmetry summaries, handling of extreme values and follow-up subsets, yearly change ranged from 3.0 to 3.2%, size-relative change from 1.5 to 1.7% and the within-child exponent from 1.61 to 1.79. Similar estimates were obtained with the vertex-to-vertex distance (3.1% per year, 95% CI 2.1 to 4.2; exponent 1.70, 95% CI 1.19 to 2.21), after excluding children with any recorded crossbite (3.1%, 95% CI 2.2 to 4.1; 361 children) or without Angle Class I occlusion at every visit (2.9%, 95% CI 1.7 to 4.0; 284 children), and when each child was left out in turn (3.05 to 3.25% per year).

Asymmetry values differed between imaging sessions (likelihood-ratio test, 10 degrees of freedom, *p* = 0.009). Because each cohort was imaged on its own schedule, elapsed time within a child was almost completely determined by session. Adjusting for session left the point estimate similar (3.4% per year) but made it imprecise (95% CI −1.4 to 8.5); this interval includes no change, so a contribution of session effects to the within-child estimate cannot be excluded. Among children imaged in the same session, older children were more asymmetric (2.6% per year of age, 95% CI 1.4 to 3.7); this comparison is between children and does not independently confirm within-child change.

The variation not explained by the model (residual variability) was about 0.04 mm in every age band, obtained by multiplying the log-scale residual SD by the band median. On the log scale, it decreased from 0.100 at 6–8 years to 0.076 at 15–18 years (exploratory Levene test, *p* = 0.012; Figure S2). This residual variation combines biological variation, variation from image capture and processing, and departures from the model, so it is not a measure of measurement error. The Levene test is only descriptive here, because repeated scans of the same child are not independent. For comparison, the estimated annual increase, about 0.013 mm at a typical asymmetry of 0.42 mm, is roughly one-third of this residual variability. The two quantities are not directly comparable, but the contrast shows that a precisely estimated population trend does not imply that such changes can be detected in an individual child.

### 3.5. Individual Examples

**Figure 5:**
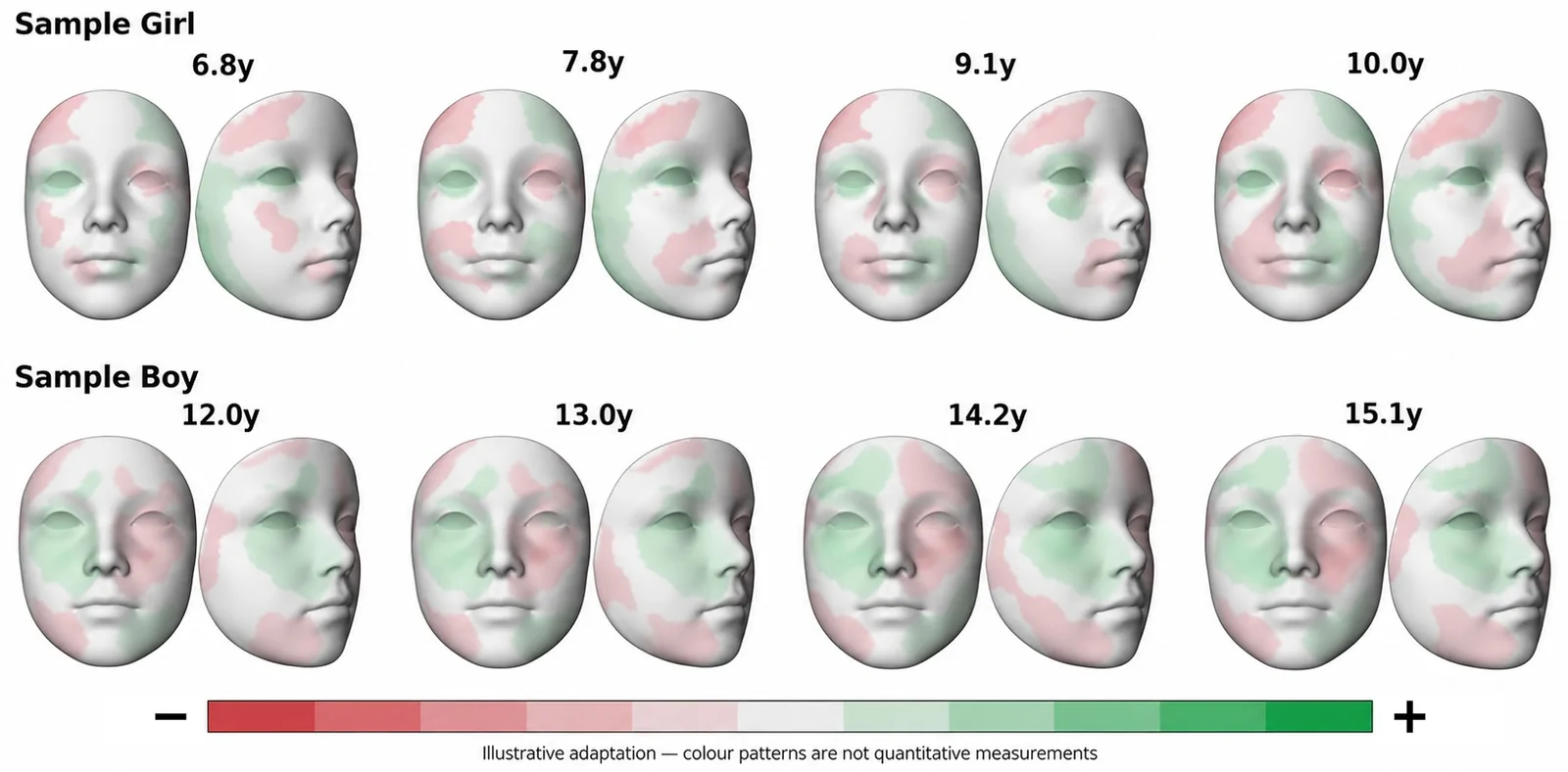
Schematic illustration of facial asymmetry patterns in two children at each of their four scans: a girl aged 6.8 to 10.0 years (**top**) and a boy aged 12.0 to 15.1 years (**bottom**), in frontal and oblique views. The colour patterns were adapted from the children’s measured asymmetry maps with a generative AI tool (ChatGPT, OpenAI) and drawn on a generic face, so that neither child can be identified. Red and green indicate opposite signs of the side difference between the facial surface and its mirror image; the colours carry no numerical values and are not measurements. The analysis itself used absolute distances on the left hemiface only.

Figure 5 illustrates how asymmetry is distributed over the face in two children at each of their four scans. It is a schematic illustration, not a quantitative display: the colour patterns were adapted from the children’s measured asymmetry maps and drawn on a generic face, so that neither child can be identified. The girl was imaged at 6.8, 7.8, 9.1 and 10.0 years (whole-face asymmetry 0.42, 0.45, 0.40 and 0.49 mm) and the boy at 12.0, 13.0, 14.2 and 15.1 years (0.49, 0.55, 0.58 and 0.55 mm). They were chosen to cover the observed age range, with individual trends close to the model estimate (2.8% and 3.7% per year, fitted across all four scans) and with asymmetry close to the sample median (girl) or the upper quartile (boy). The schematic colour patterns cannot be used to assess the magnitude of year-to-year changes in asymmetry. The two children illustrate the measure; they do not represent all children.

## 4. Discussion

In this study, the face became slightly more asymmetric as children grew: within the same child, asymmetry increased by about 3% per year over a median follow-up of 2 years, and older children were more asymmetric than younger ones by a similar amount per year of age. This estimate was consistent across the additional analyses, but when differences between scanning sessions were taken into account, it became much less certain, and the possibility of no change could no longer be excluded (Section 4.1). Over the same period, asymmetry also increased more than facial size: relative to facial size it rose by 1.6% per year, and for every 1% increase in facial size, asymmetry increased by about 1.7%. Because children’s faces grow as they get older, we could not tell whether this reflects facial growth itself or other age-related changes; when age was taken into account, the size-related estimate became very uncertain. Contrary to our hypothesis, the increase in asymmetry therefore did not simply keep pace with facial growth. Earlier studies compared asymmetry between age groups or followed it over time; to our knowledge, none has examined how asymmetry changes relative to facial size in the same growing children. Boys were more asymmetric than girls, but the difference became smaller once their larger faces were taken into account.

### 4.1. Developmental Trajectory

Our findings differ from those of small longitudinal studies reporting little or no change after early childhood [6,16,18] and agree with cross-sectional reports of greater asymmetry at older ages [17,19,20] (Table S2). Such comparisons are limited, because studies differ in how faces were imaged, whether landmarks or whole surfaces were analysed, how faces were aligned, which facial areas were included and how asymmetry was defined. For example, the percentage of surface matching within a tolerance, regional RMS distances, distances to a reference plane and perpendicular distances to a mirrored surface do not measure the same thing. A larger sample increases precision, but differences between studies cannot be explained by statistical power or measurement sensitivity alone.

Part of the yearly increase could, in principle, reflect differences between imaging sessions rather than growth. Asymmetry values differed somewhat between sessions, and because each school group was scanned on its own fixed schedule, the time since a child’s first scan was almost entirely determined by the session. When session was taken into account, the estimated yearly increase stayed about the same (3.4% per year) but became much less certain (95% CI −1.4 to 8.5), so the possibility of no change could no longer be excluded. Older children scanned in the same session were also more asymmetric than younger ones (2.6% per year of age), but this compares different children rather than following the same child. We therefore cannot rule out that small differences in scanning conditions contributed to the observed change. The yearly increase is best read as a consistent average estimate that relies on the assumption that scanning sessions did not differ systematically.

A large cross-sectional study found no association with age in 533 children aged 0–18 years [14]. One possible explanation is that it included early childhood, when Finnish longitudinal studies found asymmetry to decrease [7,9]; differences in population and measurement may also contribute. In the present study, the change observed within children over a few years was statistically compatible with the differences between children of different ages, which suggests that short-term changes are consistent with the population age pattern. This compatibility, however, does not prove that the two are equivalent or rule out differences between birth cohorts.

Whether asymmetry decreases in early childhood and then increases during school age and adolescence remains a hypothesis. Our sample included no children younger than six years, so it cannot show a minimum or turning point. Similar numerical values in different studies are not sufficient evidence, because imaging, processing and outcome definitions differ. Testing this pattern would require a cohort spanning both periods, analysed with the same validated method.

Together with adult studies [19,20], the present findings suggest that increasing surface asymmetry can occur during more than one period of life.

### 4.2. Facial Size and Sex

As children grew, facial asymmetry increased more than in proportion to facial size over the observation period. The asymmetry-to-size ratio increased over time, and the allometric analysis estimated that each 1% increase in facial size was associated with approximately a 1.73% increase in asymmetry within a child. Thus, dividing asymmetry by facial size did not fully remove its association with size. These analyses provide complementary descriptions of growth, but cannot distinguish the association with increasing facial size from other age-related changes.

Within individual children, facial asymmetry increased faster than facial size over the observed period. However, children also became older, and their stage of maturation changed. When age was included in the analysis, the estimated allometric exponent was 0.89, with a wide confidence interval (95% CI −0.13 to 1.91). We therefore could not clearly separate the association with facial size from other age-related changes. These findings do not explain why asymmetry increases during growth. Centroid size measures overall facial size; measurements of specific facial dimensions or regions may reveal additional patterns.

The difference in asymmetry between boys and girls became smaller after accounting for facial size, suggesting that facial dimensions may contribute to the difference [20,26]. However, the confidence interval for the difference in the asymmetry-to-size ratio (−2.0 to 8.6%) still allowed either greater or lower values in boys. Dividing asymmetry by facial size assumes proportional growth and may not fully account for size differences. We therefore also compared boys and girls using models that accounted for facial size and for size and age together. The estimated differences were 0.9% (95% CI −4.3 to 6.3) and 1.2% (95% CI −4.3 to 7.1), respectively. These smaller estimates do not prove that facial size explains the sex difference or that boys and girls have equal asymmetry. Similarly, we found no clear difference in their annual rates of change, but this does not prove that their growth patterns were identical. Differences in pubertal timing may influence both comparisons between boys and girls and changes within individual children.

### 4.3. Interpretation of the Asymmetry Measure

Our whole-face measure describes the amount of asymmetry without identifying which side is more prominent. It combines a consistent tendency for one side to differ from the other (directional asymmetry) with individual deviations that can occur on either side (fluctuating asymmetry). These patterns may develop differently during growth [10,11], and our analysis cannot show which contributed to the observed increase. Averaging across the face may also hide larger differences in specific areas, such as the chin, jaw, nose or tissues around the mouth. We did not analyse changes in individual facial regions.

Our method measures the perpendicular distance from points on the facial surface to the mirrored surface. This describes differences in surface prominence and is different from measuring distances between matching points on the two sides of the face. The way the surfaces are aligned, how well the template fits, and where measurement points are placed can affect the overall result. We did not independently check whether the method was equally accurate across ages and facial sizes. We also did not test alternative alignment methods or whether uniformly enlarging a face produced a proportional increase in measured asymmetry. Automatic processing alone does not guarantee these properties. Using another summary measure, root-mean-square distance (RMS), gave similar results, but this does not rule out errors shared by both measures. Reference values therefore remain specific to the imaging and processing methods used.

This study measured the soft-tissue surface of the face, not the underlying bones. Soft tissues can partly conceal skeletal asymmetry, especially where they are thicker. In areas such as the chin, the surface may more closely reflect the underlying bone [1,12]. Our findings therefore describe visible facial soft-tissue asymmetry and should not be interpreted as measurements of skeletal asymmetry.

### 4.4. Clinical Relevance

The absolute changes were small. In the descriptive age-by-sex model used for Table S3, predicted average asymmetry increased from 0.38 to 0.49 mm in girls and from 0.40 to 0.52 mm in boys between 7 and 16 years, a difference of about 0.1 mm in both sexes. These are differences between fitted population averages, not nine-year changes observed in the same children. They describe global soft-tissue surface asymmetry at the population level: they do not establish thresholds for pathological asymmetry, do not show that the change is clinically perceptible or surgically relevant, and are not diagnostic criteria. The findings provide developmental context, but monitoring individual patients would require known measurement error. The estimated annual increase (about 0.013 mm at a typical asymmetry of 0.42 mm) is well below the residual variability between scans (about 0.04 mm), so the present data support a population-level trend but do not establish the measurement resolution needed to monitor subtle change in individual patients. Diagnostic thresholds and treatment decisions would also require links to regional anatomy and to clinically meaningful outcomes. Because children with clinically visible facial asymmetry or pronounced skeletal malocclusion were excluded at screening, the values describe children without such features. They may underestimate asymmetry in unselected children and are not directly applicable as reference data for orthodontic patients, in whom asymmetry and malocclusion are more frequent.

### 4.5. Strengths and Limitations

Strengths include the relatively large mixed-longitudinal dataset, common dense surface sampling, separation of within-child and between-child components, and similar estimates across the reported sensitivity analyses.

Repeated captures were not performed, so the repeatability of the complete imaging and processing workflow could not be quantified. Automatic processing gives the same result when the same image is processed again, but this does not show that the measurement is accurate or that a new capture would give the same value. Random measurement error that averages out on the log scale would mainly reduce precision rather than create a trend over time, but this cannot be assumed for unsigned surface distances: errors may vary with age, facial size or shape. Residual diagnostics and influence checks can reveal some problems, but they cannot exclude systematic registration bias or replace repeated captures. The residual variability of about 0.04 mm includes biological and model-related variation as well as measurement variation. Session effects were present, and adjusting for them made the within-child estimate imprecise; the age association within sessions compares different children and does not independently confirm the change within children. The findings therefore describe associations at the population level, and whether yearly change can be detected in an individual child remains unknown. Symmetry results were linked to scans by the laboratory-confirmed date order rather than by unique scan identifiers; incomplete processing added uncertainty in 23 children, and excluding them gave similar results.

Individual follow-up was short (median 2.0 years; maximum 3.2), so the age pattern across the whole range relies on overlapping observations from children who entered at different ages. Only four of ten invited schools took part, and the numbers of eligible and approached children were not recorded, so selection bias cannot be excluded. Children judged at screening to have a markedly retruded chin or visible facial asymmetry were excluded; this may have removed the most asymmetric children and lowered the reference values. Follow-up was limited to three or four sessions because further sessions were cancelled during the COVID-19 pandemic. Reasons for missed visits, including withdrawal for orthodontic treatment, were not recorded, so missing data related to asymmetry cannot be ruled out. Pubertal timing and functional mandibular shift were not recorded and may also have influenced the estimates. Finally, the sample came from a single Central European population, defined by both parents being native Czech speakers, which limits generalisation.

### 4.6. Future Research

Longer follow-up with indicators of maturation would clarify the timing of these changes. Independent cohorts and quantified repeat-capture reliability are needed before the values can serve as transferable references. Longitudinal analysis of predefined facial regions, including how much each region contributes to whole-face change, would show where the increase occurs. Linking regional asymmetry to perceived appearance, function and treatment outcomes would help to judge its clinical relevance.

## 5. Conclusions

In this mixed-longitudinal sample, whole-face soft-tissue asymmetry increased modestly on average within children over short observation periods, and older children were more asymmetric than younger ones by a similar amount; after adjustment for imaging session, the within-child estimate was imprecise. Over the observation period, asymmetry increased more than in proportion to facial size; this is a descriptive longitudinal association, and the separate contributions of facial enlargement and age could not be determined. The difference between boys and girls became smaller when asymmetry was expressed relative to facial size, although uncertainty remained. The estimated annual increase (about 0.013 mm at a typical asymmetry of 0.42 mm) was well below the residual variability between scans. These population-level findings provide developmental context; monitoring individual patients and diagnostic use will require measurement validation and clinically meaningful reference criteria.

## Figures and Tables

**Table 1:** Sample characteristics.

Characteristic	All	Girls	Boys
Children, n	377	222	155
Scans, n	1014	584	430
Scans per child: 1/2/3/4	40/99/176/62	25/60/109/28	15/39/67/34
Age at first scan, y, mean (SD)	10.8 (2.6)	11.0 (2.6)	10.6 (2.7)
Age at first scan, y, range	6.0–17.5	6.4–17.5	6.0–17.5
Age at any scan, y, range	6.0–18.8	6.4–18.5	6.0–18.8
Children with ≥2 scans, n (%)	337 (89)	197 (89)	140 (90)
Follow-up in children with ≥2 scans, y, median (IQR)	2.0 (2.0–3.2)	2.0 (2.0–2.2)	2.0 (2.0–3.2)
Centroid size at first scan, mm, mean (SD)	4774 (266)	4728 (258)	4841 (264)

SD, standard deviation; IQR, interquartile range; y, years.

**Table 2:** Whole-face asymmetry and centroid size by age.

Measure	6–8 y	9–11 y	12–14 y	15–18 y
Asymmetry, mm, median (IQR)	0.40 (0.34–0.49)	0.43 (0.36–0.52)	0.46 (0.38–0.55)	0.53 (0.44–0.66)
Centroid size, mm, mean, girls/boys	4491/4639	4701/4854	4924/5054	5060/5287
Scans, n	169	404	330	111
Children, n	110	234	188	69
Consecutive scan pairs, n	150	265	180	42

IQR, interquartile range; y, years. Asymmetry summaries exclude the 11 missing whole-face values. Scan-level values; children contribute up to four scans. Children are counted in every band in which they have a scan; consecutive pairs are assigned to the band of the earlier scan. Missing whole-face outcomes by band: 1, 6, 3 and 1. Age bands: 6–8 y, 6 ≤ age < 9; 9–11 y, 9 ≤ age < 12; 12–14 y, 12 ≤ age < 15; 15–18 y, age ≥ 15.

**Table 3:** Development of absolute whole-face asymmetry.

Parameter	Estimate (95% CI)	*p*-Value
Within-child change, %/year	3.2 (2.2 to 4.1)	<0.001
Between-child difference per year of baseline age, %	2.7 (1.6 to 3.8)	<0.001
Between-child minus within-child age effect, percentage points/year	−0.5 (−1.9 to 1.0)	0.532
Boys vs. girls, %	6.5 (1.0 to 12.2)	0.020
Sex × time interaction, percentage points/year	−0.02 (−1.85 to 1.80)	0.980
Spline vs. linear age model (likelihood-ratio test)	χ^2^ = 2.07, 3 df	0.559

CI, confidence interval. Linear mixed model of log mean absolute distance with baseline age centred at 11 years, elapsed time, their interaction and sex; correlated random intercept and time slope per child. Girls are the reference group. The predicted geometric mean for girls at entry age 11 years and elapsed time zero was 0.42 mm. For centred baseline age B, annual change is 100 × [exp(β2 + β3B) − 1]. The between-child coefficient describes the baseline-age gradient at elapsed time zero, not change observed within a child. Its contrast with within-child change compares the back-transformed baseline-age gradient with the annual slope at entry age 11. Sex-by-time results come from the model with that additional interaction. Exact differences in annual change are evaluated at entry age 11 years; the baseline-age-by-time contrast is the difference in annual change between entry at 12 and at 11 years (Supplementary Methods S1). Reported contrast CIs use the delta method. The nested spline comparison used ML, identical observations and random effects; its 3 degrees of freedom equal the fixed-effect design-matrix rank difference.

**Table 4:** Size-relative whole-face asymmetry and allometric exponents.

Parameter	Estimate (95% CI)	*p*-Value
Size-relative change, %/year	1.6 (0.7 to 2.6)	<0.001
Within-child allometric exponent	1.73 (1.28 to 2.19)	0.002
Between-child allometric exponent	1.69 (1.20 to 2.18)	0.006
Boys vs. girls, size-relative, %	3.1 (−2.0 to 8.6)	0.238
Boys vs. girls at equal centroid size (allometric model), %	0.9 (−4.3 to 6.3)	0.750
Boys vs. girls adjusted for centroid size and age, %	1.2 (−4.3 to 7.1)	0.671
Within-child allometric exponent, adjusted for age	0.89 (−0.13 to 1.91)	0.832

CI, confidence interval. The size-relative outcome is log(mean absolute distance/centroid size), with the same age/time and sex structure as the primary model. Girls are the reference group. Allometric exponents are estimated in a separate model of log asymmetry using within-child and between-child log centroid size, sex and a random participant intercept. These exponents describe associations without joint age adjustment. Exponents are tested against one (isometry); percentage changes and sex differences are tested against zero. The size-and-age-adjusted sex difference and the age-adjusted exponent are from the allometric model with baseline age and elapsed time added.

## Data Availability

The data presented in this study are available on request from the corresponding author due to privacy restrictions: three-dimensional facial images are identifiable personal data of minors.

## Supplementary Materials

The following supporting information can be downloaded at: https://www.mdpi.com/article/10.3390/jcm15218583/s1, Supplementary Methods S1: Detailed statistical methods; Table S1: Sensitivity and measurement-check analyses: whole-face estimates; Table S2: The present study compared with previous 3D studies of facial asymmetry and age; Table S3: Model-predicted whole-face asymmetry (geometric mean, mm) by age and sex; Figure S1: Scan flow; Figure S2: Model diagnostics. References [3,4,6,7,9,14,16,17,19,20,22] are cited in the Supplementary Materials.

## Abbreviations

The following abbreviations are used in this manuscript:

3D	three-dimensional
CI	confidence interval
ICP	iterative closest point
IQR	interquartile range
RMS	root-mean-square
SD	standard deviation
