# Supplementary material for: Development of Facial Soft-Tissue Asymmetry During Growth: A Mixed-Longitudinal Three-Dimensional Study
Source: J Clin Med. 2026 Nov 4;15(21):8583. doi: 10.3390/jcm15218583 (PMC13763984; doi:10.3390/jcm15218583)
Supplement: Supplementary file 1 [file jcm-15-08583-s001.zip › jcm-4640960-supplementary.pdf]

Supplementary Materials

Development of Facial Soft-Tissue Asymmetry during Growth: A Mixed-Longitudinal Three-Dimensional Study

Contents

Supplementary Methods S1. Detailed statistical methods
Table S1. Sensitivity and measurement-check analyses: whole-face estimates
Table S2. The present study compared with previous 3D studies of facial asymmetry and age
Table S3. Model-predicted whole-face asymmetry (geometric mean, mm) by age and sex
Figure S1. Scan flow
Figure S2. Model diagnostics

Supplementary Methods S1. Detailed statistical methods

S1.1. Scale of analysis and reporting

Whole-face asymmetry (mean absolute perpendicular distance, mm) and centroid size (mm) were analysed after natural logarithmic transformation. Individual coefficients β were back-transformed as 100 × [exp(β) − 1]. Exact differences between annual percentage changes were calculated as differences of the relevant back-transformed slopes, with delta-method CIs using their joint coefficient covariance. The between-child minus within-child comparison contrasts the baseline-age gradient at elapsed time zero with annual within-child change at entry age 11 years. For the baseline-age interaction, a finite contrast between entry ages 11 and 12 is r(1) − r(0), whereas the local derivative at entry age 11 is 100 × β3 × exp(β2), where r(B) = 100 × [exp(β2 + β3B) − 1]. These are different quantities. The reported baseline-age-by-time contrast is the finite contrast r(1) − r(0), i.e., the difference in annual change between entry at 12 and at 11 years. The exact sex-by-time contrast is the difference between boys' and girls' annual change for entry at 11 years. p-values are from two-sided Wald tests of the delta-method estimates.

S1.2. Primary model: change in asymmetry with age

Longitudinal change was analysed with a linear mixed-effects model:

$$\log(A_{ij}) = \beta_0 + \beta_1 \cdot B_i + \beta_2 \cdot t_{ij} + \beta_3 \cdot B_i \cdot t_{ij} + \beta_4 \cdot S_i + u_{0i} + u_{1i} \cdot t_{ij} + e_{ij}$$

where A_{ij} is whole-face asymmetry of child i at visit j; B_i is age at the first eligible scan, centred at 11 years; t_{ij} is time since the first eligible scan in years; S_i indicates boys (girls are the reference group); u_{0i} and u_{1i} are child-specific random intercept and slope with an unstructured covariance; and e_{ij} is the residual. Estimates were obtained by restricted maximum likelihood (REML); likelihood-ratio tests used maximum likelihood (ML). The estimated random-effect SDs were 0.25 (intercept) and 0.036 per year (slope), with correlation −0.18.

This parameterization separates within-child change (β_2) from between-child differences in baseline age (β_1). Because B is centred at 11 years, β_2 is the annual within-child change for a child entering the study at 11 years. With the baseline-age-by-time interaction, the annual change for a child entering at centred baseline age B is 100 × [exp(β_2 + β_3B) − 1]; for example, 3.3% (95% CI 1.5 to 5.2) for entry at 6 years and 3.0% (95% CI 0.3 to 5.7) at 17 years. β_1 describes baseline-age differences at elapsed time zero. Effect modification by sex was assessed with a sex-by-time interaction added to the primary model.

S1.3. Test of a constant rate of change

Departure from the specified linear age pattern was assessed by replacing elapsed time with a natural cubic regression spline of age at imaging with four degrees of freedom (Patsy cr, quantile-based knots and a centring constraint), while retaining baseline age, the baseline-age-by-time interaction, sex and the random effects. The primary fixed-effect space is nested within the spline model because the spline spans linear age and age at imaging equals baseline age plus elapsed time. The reported ranks were 5 and 8, with combined rank 8. Both models used ML on the same observations and the same random-effects structure; the rank difference gave 3 test degrees of freedom. The likelihood-ratio statistic was 2.07 (p = 0.559). This comparison concerns the specified spline alternative and does not establish a constant trajectory. The spline term was specified in Patsy as cr(age, df=4, constraints="center"), with boundary knots at the minimum and maximum age at imaging (6.01 and 18.80 years) and internal knots at the quartiles of age at imaging (9.63, 11.53 and 13.45 years). Both fits converged.

S1.4. Model diagnostics

Diagnostics comprised residuals against fitted values, residual SD by age band, normal quantile plots of residuals and random effects, and leave-one-child-out changes in the within-child estimate (Figure S2).

Approximate residual SD in millimetres was obtained by multiplying each log-scale residual SD by the band median asymmetry. This approximation does not estimate technical measurement error. The reported Levene test is treated as exploratory because repeated observations and fitted residuals are not independent raw samples. Residual variability includes biological variation, acquisition and processing variation, and model misspecification. Residuals were conditional residuals (observed values minus fitted values including the child-specific random effects). The Levene test used median centring (Brown–Forsythe variant). All 376 leave-one-child-out refits converged.

S1.5. Asymmetry relative to facial size

Analyses of facial size proceeded in three steps.

- Annual within-child change in centroid size was estimated with the primary model structure, using log centroid size as the outcome.
- The ratio of asymmetry to centroid size was analysed as log(A/CS) with the primary model structure. The ratio is dimensionless because both quantities share the same length unit, and it imposes an exponent of one (proportional scaling). A null time slope for the ratio would be consistent with proportional scaling; a ratio analysis is not equivalent to estimating an allometric exponent.
- Allometric exponents were estimated by modelling log asymmetry on each child's mean log centroid size (between-child component) and each observation's deviation from that mean (within-child component), together with sex and a random participant intercept. Both exponents were tested against one (isometry). In this model, the sex coefficient compares girls and boys with equal child-specific mean log centroid size and equal deviation from that mean.

A sensitivity model added a random slope for the within-child centroid-size component. A further model added baseline age and elapsed time, without their interaction, to the allometric predictors (1,003 observations, 376 children). Its sex coefficient compares children conditional on both size and age. The within-child correlation of time and log centroid size was calculated after centring time within each child ($r = 0.90$). VIFs of 3.4 and 3.3 refer to the elapsed-time predictor and within-child log-size component in the joint fixed-effect design matrix; elapsed time was not child-mean-centred in that matrix. These statistics therefore describe different parameterizations. Each child's mean log centroid size was calculated over the scans entering the model, i.e., scans with non-missing whole-face asymmetry.

S1.6. Sensitivity analyses

The following analyses were performed (results in Table S1):

- root-mean-square (RMS) instead of mean absolute distance;
- isolated extreme single-point values (any point beyond ±10 mm) retained, and all 12 flagged scans excluded;
- children with at least two eligible scans;
- children with complete symmetry series, meaning every dated scan had symmetry results; this addresses incomplete processing/linkage coverage and does not imply attendance at every scheduled visit or independently validate the linkage;
- Euclidean distance between each point and its mirrored counterpart (vertex to vertex) instead of the perpendicular distance;
- exclusion of children with any recorded crossbite, and restriction to children with Angle Class I at every recorded visit;

- within-child change estimated separately for baseline age <10, 10–12.9 and ≥13 years, using a model with stratum-specific intercepts and time slopes, sex, and a random intercept and time slope per child; baseline age was not included, so each slope is the average within-child change in that stratum;
- imaging session (11 sessions) added as fixed effects, tested with an ML likelihood-ratio test (10 degrees of freedom); because elapsed time and session are closely linked, an additional model used age at imaging, sex and session fixed effects with a random intercept per child to describe the age gradient among children imaged in the same session; this is not the primary within-child estimate;
- leave-one-child-out refits of the primary model (376 refits).

Baseline age and elapsed time were defined from the first eligible scan. This was the first scan with symmetry results that passed processing, identified before outcome-specific masking of isolated extreme values; the time origin was not recomputed in sensitivity subsets. The operator for individual scans was not recorded, and only left-hemiface outputs were exported, so those checks could not be performed. Alternative alignment and isotropic-scaling validation were not undertaken. Deterministic processing of an unchanged input would not answer those validation questions.

S1.7. Missing observations

Likelihood-based mixed-effects inference uses all available observations and is valid under an adequately specified outcome model and missingness at random conditional on the information included in the analysis. Reasons for missed visits were not recorded, and absence related to orthodontic treatment, which ended participation under the study protocol, could not be distinguished from other causes. Because the missing-at-random assumption concerns unobserved values, informative missingness cannot be excluded.

S1.8. Descriptive age predictions (Table S3, Figure 3)

Table S3 and the fitted curves in Figure 3 come from a separate mixed model of log asymmetry with age at imaging, sex and their interaction, and a random intercept and age slope per child. This model does not separate within-child change from between-child age differences. Its geometric-mean predictions, with 95% CIs from the fixed-effect covariance, describe the population age pattern; they are not estimates from the primary model and not prediction intervals for individual children.

S1.9. Software

Analyses were performed in Python 3.11 (original analyses) and 3.13 (revision analyses) using statsmodels version 0.15.

Table S1. Sensitivity and measurement-check analyses: whole-face estimates.

Analysis	Children	Scans	Annual change or other specified result (95% CI)	Size-relative change, %/year (95% CI)	Within-child exponent (95% CI)
Sensitivity analyses					
Primary analysis	376	1,003	3.2 (2.2 to 4.1)	1.6 (0.7 to 2.6)	1.73 (1.28 to 2.19)
Root-mean-square instead of mean absolute distance	376	1,003	3.1 (2.1 to 4.0)	1.5 (0.6 to 2.4)	1.65 (1.20 to 2.10)
Isolated extreme-point values retained	377	1,014	3.2 (2.2 to 4.2)	1.6 (0.7 to 2.6)	1.79 (1.31 to 2.27)
All 12 flagged scans excluded	375	1,002	3.2 (2.2 to 4.1)	1.6 (0.7 to 2.6)	1.73 (1.28 to 2.19)
Children with ≥2 eligible scans	336	963	3.2 (2.3 to 4.2)	1.7 (0.7 to 2.6)	1.73 (1.28 to 2.19)
Complete series (every dated scan with symmetry results)	353	956	3.0 (2.1 to 4.0)	1.5 (0.5 to 2.4)	1.61 (1.15 to 2.07)
Vertex-to-vertex (Euclidean) distance	376	1,013	3.1 (2.1 to 4.2)	1.6 (0.5 to 2.6)	1.70 (1.19 to 2.21)
Children with any recorded crossbite excluded	361	961	3.1 (2.2 to 4.1)	–	–
Children with Angle Class I at every recorded visit	284	743	2.9 (1.7 to 4.0)	–	–
Imaging-session fixed effects (11 sessions)	376	1,003	3.4 (−1.4 to 8.5)	–	–
Between-child age gradient within imaging sessions	376	1,003	2.6 (1.4 to 3.7)	–	–
Baseline age <10 years	150	438	2.9 (1.6 to 4.2)	–	–
Baseline age 10–12.9 years	153	408	3.5 (2.1 to 5.0)	–	–
Baseline age ≥13 years	73	157	3.2 (0.6 to 5.8)	–	–
Random slope for within-child centroid size	376	1,003	–	–	1.82 (1.29 to 2.35)
Baseline age and elapsed time added to the allometric model	376	1,003	–	–	0.89 (−0.13 to 1.91)
Leave-one-child-out refits, range	375	–	3.05 to 3.25	–	–
Measurement checks and other tests					
Session effects, likelihood-ratio test	376	1,003	χ^2 = 23.7, 10 df, p = 0.009	–	–
Residual SD of primary model (log scale), 6–8 / 9–11 / 12–14 / 15–18 y	376	1,003	0.100 / 0.094 / 0.082 / 0.076	–	–
Residual SD, approximate mm (× band median)	376	1,003	0.040 / 0.041 / 0.038 / 0.040	–	–
Exploratory Levene test of residual variance by age band	376	1,003	F = 3.66, p = 0.012	–	–

Estimates from the primary model structure unless stated. Size-relative change: log(asymmetry/centroid size). Exponent: within-child allometric exponent from the model with within-child and between-child log centroid size and sex. Baseline age and elapsed time are defined from the first eligible scan in all analyses. "All 12 flagged scans excluded" removes every scan with an isolated point beyond ±10 mm in either distance outcome, including one flagged only in the vertex-to-vertex outcome. Session fixed effects: elapsed time is closely linked to session, so the within-child estimate is imprecise; the age gradient within sessions compares children imaged in the same session and is not a within-child estimate. Leave-one-child-out: range of the within-child estimate across 376 refits, each omitting one child. The operator for individual scans was not recorded and right-hemiface outputs were not exported, so these analyses were not possible. Residual SD includes biological, measurement and model-related variation; conversion to mm is approximate and is not technical measurement error. The Levene test is exploratory because repeated observations and fitted residuals are dependent. Repeat captures, alternative alignment validation and isotropic-scaling validation were not performed. Complete series refers to processing coverage, not attendance at every scheduled visit.

Table S2. The present study compared with previous 3D studies of facial asymmetry and age.

Study	Design	Sample and age	Acquisition	Registration / alignment	Asymmetry measure	Facial region	Reported age effect
Present study	Mixed-longitudinal; up to four annual scans	377 children overall; primary asymmetry analysis: 376 children, 1,003 scans; ages 6.0–18.8 years; median follow-up 2.0 years	VECTRA M3 stereophotogrammetry	Dense template (7,160 points; MeshMonk); robust Procrustes alignment to mirrored copy	Mean absolute perpendicular distance to mirrored surface (left hemiface)	Whole face	Within-child +3.2%/year (95% CI 2.2–4.1); no evidence of departure from the specified linear model (p = 0.56)
Launonen et al. 2020 [9]	Prospective cohort; two time points	75 children (35 girls); 1.0 and 3.0 y	3dMDhead (5-pod) stereophotogrammetry	Mirrored surface superimposed on original (best fit, Rapidform 2006) on the area above subnasale	Average distance (mm) and symmetry percentage between original and mirrored surfaces; landmark distances to midline	Four areas: upper face, upper and lower mid-face, lower face	Upper-face symmetry improved from 1 to 3 y ($p < 0.05$); no surface-based change in other areas
Launonen et al. 2023 [7]	Prospective population-based birth cohort; three time points	102 enrolled, 70 analysed; 1, 3 and 6 y	3dMD stereophotogrammetry	Images scaled to mean centroid size; mirrored surface superimposed on original by ICP (Rapidform)	Average distance (mm) and symmetry percentage (area within 0.5 mm); landmark distances to midline	Whole face and four facial areas	Symmetry improved from 1 to 6 y in all facial areas (whole face 0.43 mm at 6 y)
Primožič et al. 2012 [16]	Prospective longitudinal; five time points (baseline, +18, +30, +42, +54 months)	27 Slovenian kindergarten children (15 boys) without malocclusion; 5.4 y at baseline, followed for 4.5 y	3D laser surface scanning	Face superimposed on its mirror image	Average distance (mm) between face and mirror image; percentage of surface not coinciding within 0.5 mm; colour maps	Whole face; upper, middle and lower face	No significant change over 54 months; no sex difference; whole-face distance 0.22–0.85 mm; upper face least asymmetric
Djordjevic et al. 2013 [6]	Prospective longitudinal; three time points (baseline, +2.5 y, +4.5 y)	60 Finnish adolescents (30 boys) at mean 11.5 y; 39 completed (mean 16 y)	Two laser scanners (Konica Minolta VI-900)	Mirror shell superimposed on original (best-fit registration) to define midsagittal plane; in-house subroutine	Surface matching (% of area within 0.5 mm) of original and mirror shells; 21-landmark linear and angular measures	Whole face and thirds	No significant change over 4.5 y in either sex; no sex difference
Hřebcová et al. 2026 [22]	Semi-longitudinal (annual scans 2021–2024), analysed as three independent age groups	46 right-handed Czech adolescents (24 boys); 15–20 y (groups 15–16, 17–18, 19–20 y)	3dMDface stereophotogrammetry	11 landmarks; non-rigid dense mesh registration and GPA (Morphome3cs); mirrored mesh aligned to original	Directional asymmetry: vertex-wise original-to-mirror distances (form and shape); MANOVA of individual-asymmetry PC scores	Whole face (vertex-wise maps)	No significant effect of age group or sex on global directional asymmetry; localized changes only
Cho et al. 2018 [14]	Cross-sectional	533 children, 0–18 y	3D stereophotogrammetry	Symmetric 3D template fitted to each scan using 25 landmarks	Distances between paired left and right template points	Head and face, reported separately	No significant differences by age, sex or race; facial asymmetry 1.2 ± 0.6 mm, head 1.5 ± 0.5 mm
Hsu et al. 2019 [3]	Cross-sectional	652 Taiwanese elementary-school children; 6–12 y	3dMD stereophotogrammetry	32 manual landmarks; symmetric template deformed to each image (thin-plate spline and closest-point matching)	Mean distance between paired left and right template points	Face and skull, reported separately	Age effect not tested statistically; side differences in average head models "appeared to increase with age"; facial asymmetry 0.96 ± 0.53 mm, no sex difference
Cappella et al. 2023 [17]	Cross-sectional; two age classes	135 Italian children (74 boys); 4–8 and 9–12 y	VECTRA M3/H1 stereophotogrammetry	Mirrored model registered on original (ICP; VAM 5.3.1)	RMS point-to-point distance between original and mirrored models	Facial thirds	Older children more asymmetric ($p < 0.001$); boys more asymmetric (p = 0.005); RMS 0.30–0.51 mm
Linden et al. 2018 [19]	Cross-sectional	191 subjects; 0.3–88 y (mean 26.7)	Canfield Vectra stereophotogrammetry	Reflection about the plane of maximal symmetry (best fit of native and reflected faces)	RMS deviation between original and reflected faces	Whole face and thirds	Positive linear association with age (r = 0.66, $p < 0.001$), about 0.06 mm per decade; increase in all thirds
Verhoeven et al. 2024 [20]	Cross-sectional; 15 age groups	600 Dutch subjects (300 male); 0 to 70+ y	3dMDface two-pod stereophotogrammetry	Landmark pre-alignment and adapted ICP of mirrored image	Absolute mean distance between original and mirrored surfaces	Whole face	Increase from 0.45 mm (0–4 y) to 0.98 mm (70+ y); r = 0.55, $p < 0.001$; males 0.77 vs. females 0.67 mm ($p < 0.001$)
Harnádková et al. 2023 [4]	Cross-sectional; three age groups	300 Czech adults (151 female); 20–80 y	3dMDface stereophotogrammetry	GPA of original and reflected-relabelled configurations of 28 landmarks (MorphoJ)	Shape directional asymmetry (Procrustes ANOVA, PCA)	Whole face (landmark configuration)	Directional asymmetry in all groups; sex differences before 40 y; largest in youngest men; widest variability in oldest group

Comparisons are qualitative because study designs, age coverage, anatomical regions, acquisition systems, alignment procedures and asymmetry definitions differ. Point-to-surface distances, bilateral point distances, thresholded surface matching and regional RMS measures are not interchangeable.

GPA, generalised Procrustes analysis; ICP, iterative closest point; PC, principal component; RMS, root mean square.

Studies of children with craniofacial anomalies, including their control groups, are not included.

Table S3. Model-predicted whole-face asymmetry (geometric mean, mm) by age and sex.

Age, years	Girls, mm (95% CI)	Boys, mm (95% CI)
6	0.37 (0.34–0.39)*	0.39 (0.37–0.42)
7	0.38 (0.36–0.40)	0.40 (0.38–0.43)
8	0.39 (0.37–0.41)	0.42 (0.39–0.44)
9	0.40 (0.38–0.42)	0.43 (0.41–0.45)
10	0.41 (0.40–0.43)	0.44 (0.42–0.46)
11	0.42 (0.41–0.44)	0.45 (0.44–0.47)
12	0.44 (0.42–0.45)	0.47 (0.45–0.49)
13	0.45 (0.44–0.47)	0.48 (0.46–0.50)
14	0.47 (0.45–0.48)	0.49 (0.47–0.52)
15	0.48 (0.46–0.50)	0.51 (0.48–0.53)
16	0.49 (0.47–0.52)	0.52 (0.49–0.55)
17	0.51 (0.48–0.54)	0.54 (0.50–0.57)
18	0.53 (0.49–0.56)	0.55 (0.51–0.59)

Predicted geometric-mean whole-face asymmetry from a separate mixed model of log asymmetry with age at imaging, sex and their interaction, and a random intercept and age slope per child (1,003 scans, 376 children). This descriptive model differs from the primary baseline-age/elapsed-time model and does not separate within-child change from between-child age differences. Values describe the fitted population mean with 95% CIs from the fixed-effect covariance; they are not individual predictions. Observed ages: girls 6.4–18.5 y, boys 6.0–18.8 y. *The estimate for girls at age 6 is a small extrapolation below their youngest observed age (6.4 years).

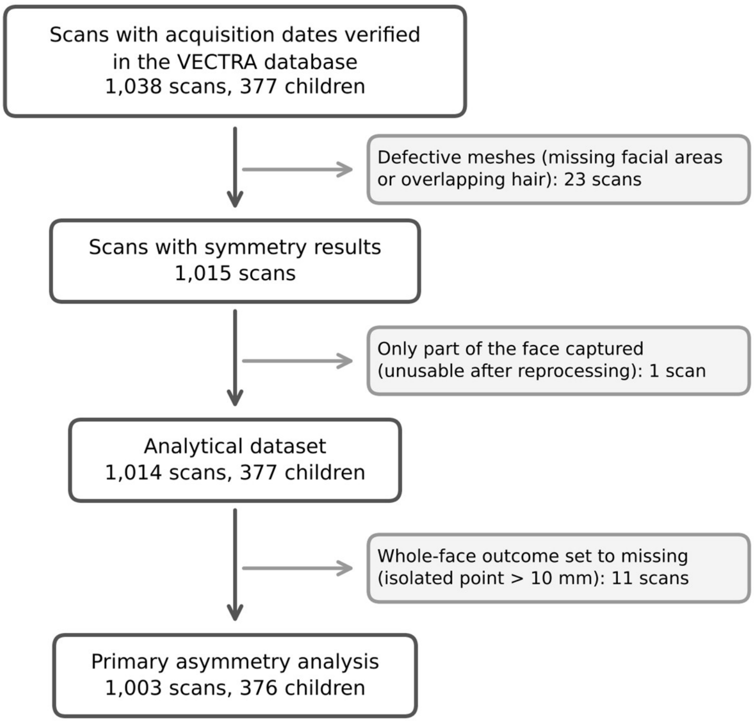

Figure S1. Scan flow. Numbers of scans and children from all scans with acquisition dates verified in the VECTRA database to the primary asymmetry analysis. The numbers of eligible and approached children were not recorded, so recruitment is not shown. In the 11 scans with an isolated point beyond 10 mm, only the whole-face asymmetry outcome was set to missing; these scans remained in the analytical dataset.

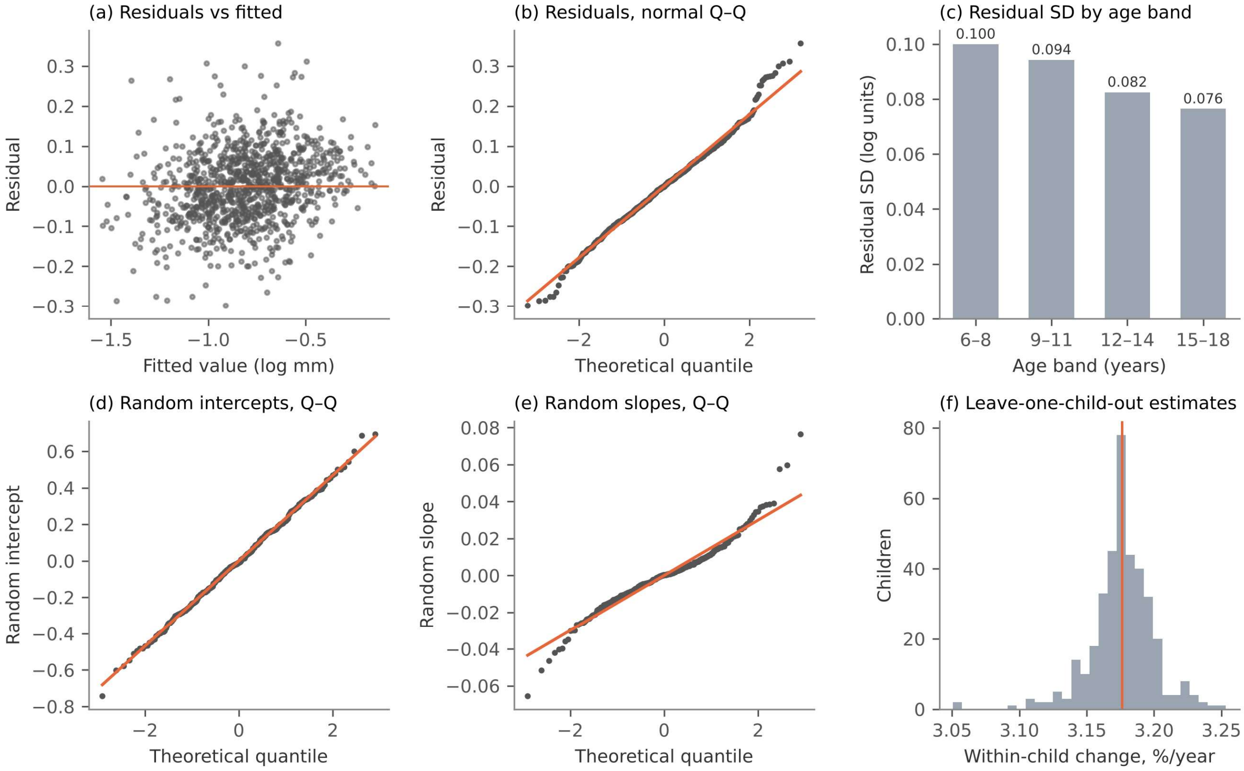

Figure S2. Model diagnostics for the primary mixed model of log whole-face asymmetry (1,003 scans, 376 children). (a) Conditional residuals (observed minus fitted values, including child-specific random effects) against fitted values. (b) Normal quantile plot of residuals. (c) Residual SD by age band on the log scale; multiplied by the band median asymmetry, each corresponds to approximately 0.04 mm. Residual variability combines biological variation, acquisition and processing variation and model misspecification; it is a model diagnostic, not an estimate of measurement error. (d, e) Normal quantile plots of the child-specific random intercepts and random time slopes. (f) Within-child annual change when each child was omitted in turn (376 refits, all converged; range 3.05–3.25% per year); the vertical line marks the full-sample estimate.
